# What Is Pyorrhea?

**Published:** 1917-01

**Authors:** 


### WHAT IS PYORRHEA?

*To the Editor:*—Kindly advise me whether a physician and surgeon, regularly licensed under the Illinois law and thereby empowered to practice medicine and surgery in all their branches, requires a license as a dentist before he may treat pyorrhea alveolaris.—William A. Lurie, M. D., Chicago.

Answer.—The Illinois medical practice act defines the practice of medicine as treating or professing to treat, operating on or prescribing for any physical ailment or any physical injury to or deformity of another. The Illinois dental law defines dentistry as treating or professing to treat any of the diseases or lesions of the human teeth or jaws, or extracting teeth, or preparing or filling cavities in human teeth, or correcting the malposition of teeth, or supplying artificial teeth as substitutes for natural teeth Where is the dividing line between medicine and dentistry? In 1899, the Rhode Island Supreme Court (*State* v. *Beck*, 21 R. I. 288, 43 Atl. 366) held that a legally qualified physician could practice dentistry. The court said:

''Dentistry is now a well-recognized branch of surgery. A dentist is a dental surgeon. He performs surgical operations on the teeth and jaw and as incidental thereto upon the flesh connected therewith. The sphere of operations, then, is included in the larger one of the physician and surgeon. A fair and reasonable construction of the two statutes taken together, therefore, comes to this, that the General Assembly * * * intended to except physicians and surgeons from the restrictions imposed on other persons regarding the practice of dentistry. * * * It has always been the custom in this state and probably everywhere else for physicians to treat ailing teeth, to extract teeth and to perform various other professional services which technically come under the purview of dentistry. * * * To now prohibit them from thus treating their patients would be * * * interference with their proper and legitimate functions."

In 1909, the Minnesota Supreme Court held (*State* v. *Taylor*, 106 Minn. 218, 118 N. W. 1012) that a person licensed to practice medicine and surgery can not by virtue thereof practice dentistry without securing a license as a dentist. In this case, the defendant extracted teeth, took impressions in wax of the mouth and cavities, secured artificial teeth from dental laboratories and delivered them to patients. The court held that in the absence of any legislation to the contrary, a license to practice medicine and surgery would include dentistry, but that for reasons of public policy, the legislature had made a separate profession of dentistry. The acts of the defendant came clearly within the definition of dentistry. The legislature evidently intended to restrict the practice of the physician and surgeon and to require him, if he desires to practice dentistry, to obtain a license from the state board of dental examiners.

The question still remains whether pyorrhea is a purely local disease, involving only the teeth and gums, or whether it is a local evidence of a general and constitutional condition. Even if it is conceded that it is a purely local condition, is it a disease of the teeth or of the bone? If it is regarded as a disease of the bone, would the board of dental examiners take the position that all diseases of the jaw bone come exclusively within the realm of dentistry? If so, then the surgeon who treats a gunshot wound or a fracture of the jaw or who removes an osteosarcoma, or the rhinologist who drains an infected superior maxillary sinus, must first secure a license to practice dentistry. If the dental law forbids the physician to use local treatment for pyorrhea, does not the medical law prohibit the dentist from using general treatment for the same condition? Must the patient with pyorrhea have a dentist on one side for local treatment and a physician on the other to administer general treatment? This reduces the question to an absurdity. The right of the dentist under the Illinois statute to the extracting of teeth, the filling of cavities, the correction of malpositions and the supplying of artificial teeth will probably not be questioned. The controversy will center around that part of the statutory definition of dentistry which includes "treating any of the diseases or lesions of human teeth or jaws." If this is interpreted literally by the courts, it can only lead to confusion.—*Journal A. M. A.*, November 11, 1916.

